# Sequential solvent extraction for the modes of occurrence of selenium in coals of different ranks from the Huaibei Coalfield, China

**DOI:** 10.1186/1467-4866-8-14

**Published:** 2007-12-20

**Authors:** Ying Zhang, Guijian Liu, Chen-Lin Chou, Lei Wang, Yu Kang

**Affiliations:** 1 CAS Key Laboratory of Crust-Mantle Materials and the Environment, School of Earth and Space Sciences, University of Science and Technology of China, Hefei 230026, China; 2 Illinois State Geological Survey (Emeritus), Champaign, IL 61820, USA

## Abstract

Forms of selenium in bituminous coal, anthracite, and cokeite (natural coke) from Huaibei Coalfield, Anhui, China, have been determined by sequential solvent extraction. The selenium content in bulk samples is 4.0, 2.4, and 2.0 μg/g in bituminous coal, anthracite, and cokeite, respectively. The six forms of selenium determined by six-step solvent extraction are water-leachable, ion-exchangeable, organic matter-associated, carbonate-associated, silicate-associated, and sulfide-associated. The predominant forms of selenium in bituminous coal are organic matter-associated (39.0%), sulfide-associated (21.1%), and silicate bound (31.8%); these three forms account for 92% of the total. The organic matter bound-selenium decrease dramatically from bituminous coal (39.0%) to anthracite (11.6%) and to cokeite (0%), indicating that organic matter bound selenium is converted to other forms during metamorphism of the coal, most likely sulfide-form. The sulfide-associated form increased remarkably from bituminous coal (21.1%) to anthracite (50.4%) and cokeite (54.5%), indicating the formation of selenium sulfide, possibly in pyrite during the transformation of bituminous coal to anthracite and cokeite. The silicate-associated selenium in bituminous coal (31.8%) is much higher than that in anthracite (16.4%) and cokeite (15.8%), indicating that silicate-associated selenium is partly converted to sulfide during metamorphism.

## 1. Introduction

China is the largest coal producer and consumer in the world. It has been estimated that more than 75% of the energy production in China is based on coal and more than 400 million people in China rely on coal for their domestic energy needs [1,2]. Due to the limited petroleum and natural gas reserves, and huge coal reserve in China, coal may remain to be a dominant energy source in China for many years to come [3-6]. Unfortunately, coal utilization may bring with it environmental and human health costs [7-9]. Many of the environmental and health problems attributed to coal combustion are due to mobilization of potentially toxic elements [7-11].

Selenium (Se) is one of the volatile elements in coal. During mining and utilization of coal Se is largely released into the environment with potential environmental and human health impacts [12-16]. There was massive waterfowl aberrance and death that happened in Kesterson Reservoir of California, and selenium-poisoning of the residents in Yutangba, Enshi, Hubei, China which provided impetus for study envitronmental geochemistry of Se. Although selenium metal has little toxicity, the selenide, selenite, and other selenium compounds such as selenium fluoride have a high toxicity. Because of high toxicity of Se and its compounds, Se is detrimental to plants and animals [9]. Human selenosis in some areas in China is attributed to the practice of using high-Se combustion residues as a soil amendment [7,17,18]. It is desirable to reduce Se content in coals prior to combustion. Selenium in coal is regarded as a source of Se for soils and plants which have caused selenosis in some areas of Se-rich coal and carbonaceous shale[7,9,19-24].

The mode of occurrence of an element is a description of the manner in which an element is chemically bound in the host material. In coal, elements can be associated with the inorganic constituents or with the organic constituents. The element's modes of occurrence can strongly influence its behavior during coal cleaning, weathering, leaching, combustion, and conversion [25,26]. These different modes of occurrence will cause the element to behave differently during coal cleaning and coal combustion and thus will have different environmental and human health impacts. Detailed knowledge of the forms of Se in coals is needed for a better understanding its behavior during coal processing and utilization, and consequent environmental impacts[18,27]. In this paper we studied the forms of Se in different ranks of coals from Huaibei coalfield, Anhui Province, China, by sequential solvent extraction.

## 2. Samples and Methods

### 2.1 Geological Setting

**Figure 1:**
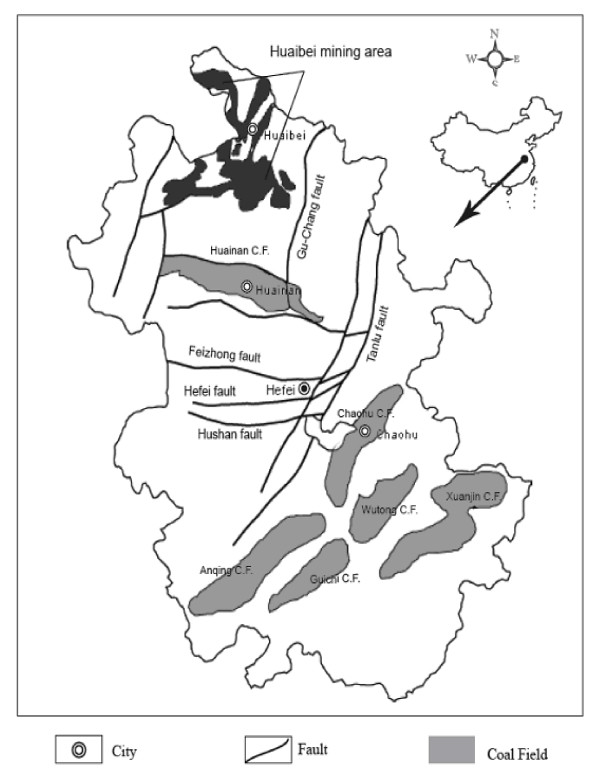
Location of the Huaibei Mining District in Anhui Province, China.

**Figure 2:**
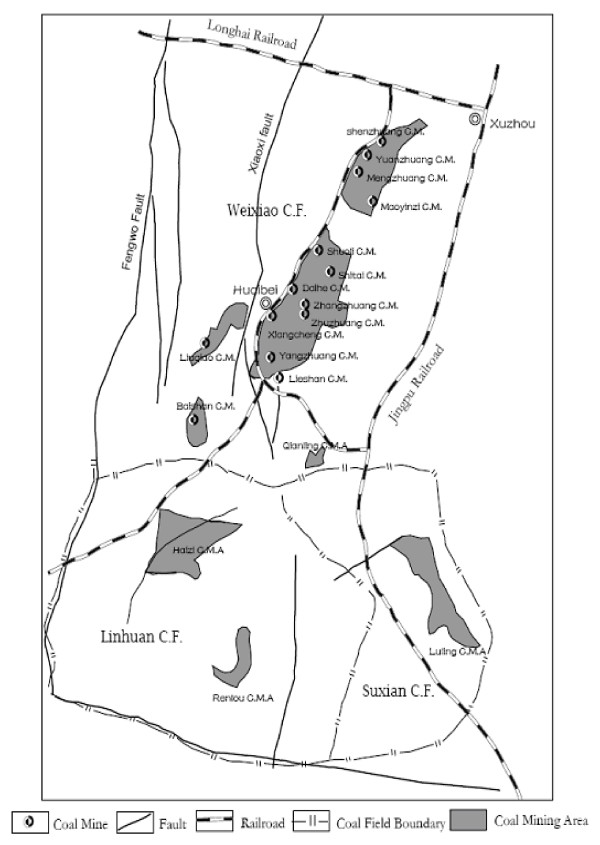
Location of the coal mines in Huaibei mining district.

The Huaibei Coalfield is located in the northern Anhui province, China (Fig. 1 and Fig. 2) with longitude at E115°58' to E117°12' and latitude at N33°20' to N34°28'. The Huaibei coalfield has an area of approximately 9,600 km^2^, in which 4,100 km^2 ^are covered by Carboniferous and Permian coal-bearing strata. The Huaibei coalfield is unique because it is intruded by igneous bodies, and the coal is metamorphosed to a broad range of coal ranks from bituminous coal to anthracite and to cokeite (natural coke) by the influence of igneous heat source [28]. The annual production of the Huaibei Coalfield is over 30 Mt, which is mainly used for power generation and industrial boilers.

### 2.2 Sample collection and preparation

We collected samples of bituminous coal, anthracite and cokeite (natural coke) from Huaibei Coalfield. The samples were stored immediately in bags to prevent contamination and weathering. The samples were air-dried, milled and split until a representative sample of 0.5 kg was pulverized to pass the 100-mesh sieve and dried for 12 hours in a desiccator. The samples were analyzed for proximate and ultimate compositions at the Energy Institute, the Pennsylvania State University.

### 2.3 Sequential extraction procedure

The sequential extraction procedure was originally developed to analyze soil samples [29], and it is later modified for analyzing coal [26,30]. A variety of sequential extraction procedures have been successfully applied to coal [31-35]. Based on these studies, we devised a sequential extraction procedure for determining the forms of selenium in coals. The six-step sequential extraction procedure is outlined in Table 1. The forms of selenium in coals may be determined as water-leachable, ion-exchangeable, organic matter-associated, carbonate-associated, silicate-associated, and sulfide-associated.

**Table 1:** Sequential chemical extraction procedure

**Form of selenium**	**Extraction method**
Water-leachable	A 3-g sample of coal was transferred into a container. Add 20 mL of Milli-Q water at room temperature. Let it stand for 24 h. The suspension was then centrifuged at 3500 rpm for 20 min.
Ion-exchangeable	20 mL of ammonium acetate (1 N) was added to the dried residual solids at room temperature for 24 h, suspension was then centrifuged at 3500 rpm for 20 min.
Organic matter-bound	The dried residual solid was treated with CHCl_3 _(1.47 g/cm^3^). After 24 h of stirring at room temperature, a float-sink separation was performed by centrifugation (3500 rpm for 20 min). The float fraction (1.47 g/cm3) of the sample was dried at 40°C, then treated with 12 mL of an oxidizing mixture (HNO_3_: HCl = 3:1) and 3 mL HF in a Telfon beaker which was put in a microwave oven (at 600 W for 30 min; at 1200 W for 25 min; and at 1200 W for 30 min).
Carbonate-bound	The sink fraction (1.47 g/cm^3^) was dried at 40°C, and then treated with 20 ml HCl (0.5%). The suspension was then centrifuged at 3500 rpm for 20 min.
Silicate-bound	The dried residual solid was treated with CHBr_3 _(2.89 g/cm^3^). After 24 h stirring at room temperature, a float-sink separation was performed by high-speed centrifugation (5000 rpm for 20 min). The float fraction (2.89 g/cm^3^) of the sample was dried at 40°C, and then treated with 12 mL of an oxidizing mixture (HNO_3_: HCl = 3:1) and 3 mL HF in a Telfon beaker which ws put in a microwave oven (at 600 W for 30 min; at 1200 W for 25 min; and at 1200 W for 30 min).
Sulfide-bound	The sink fraction (2.89 g/cm^3^) was dried at 40°C, and then treated with an oxidizing mixture (HNO^3^: HCl = 3:1) and HF in a Telfon which was put in a microwave oven (at 600 W for 30 min; at 1200 W for 25 min; and at 1200 W for 30 min).

## 3. Results and discussion

### 3.1 Proximate and ultimate analysis

The results of proximate analyses (ash, moisture, volatile matter, and fixed carbon) and ultimate analyses (C, H, N, S, ash) are listed in Table 2. These data were designed to provide information on the technological performance of coal. The carbon content increases with increasing coal rank.

**Table 2:** Proximate and ultimate analyses and total Se concentrations of different ranks of coals

	%	Bituminous coal	Anthracite	Cokeite
Ultimate analyses	Carbon	78.1	81.3	86
	Hydrogen	4.64	3.52	1.77
	Nitrogen	0.92	0.95	0.56
	Sulfur	0.48	0.25	0.48
Proximate analyses	Moisture	1.26	2.15	5.41
	Volatile matter	24.64	12.06	4.75
	Ash	14.51	13.14	6.94
	Fixed carbon	59.57	72.69	82.58

### 3.2 Results of sequential solvent extraction

The results of sequential extraction of selenium from three coals from Huaibei coalfield are listed in Table 3. The selenium content in the bulk sample of bituminous coal, anthracite and cokeite are 4.0, 2.43, and 2.0 μg/g, respectively. The total recoveries of selenium from the six solvent extraction steps are 68.9%, 79.5%, and 82.1%, which are acceptable considering cumulative errors of extraction steps and analytical errors in the determination of Se in six fractions. The selenium content decreases with increasing rank of the three coals. There is a possibility that selenium was partially lost during metamorphism.

**Table 3:** Abundance of six forms of Se in sequential extraction fractions of the bulk sample (in μg/g) and fraction of each form of Se in three coals (in %)

	Bituminous coal		Anthracite		Cokeite	
	Content (μg/g)	Fraction (%)	Content (μg/g)	Fraction (%)	Content (μg/g)	Fraction (%)

Water-leachable	ND	0.00	0.063	3.23	0.057	3.45
Ion-exchangeable	0.128	4.66	0.138	7.14	0.186	11.3
Organic matter-bound	1.08	39.1	0.219	11.3	ND	0.00
Carbonate-bound	0.093	3.36	0.224	11.6	0.245	14.9
Sulfide-bound	0.581	21.1	0.975	50.4	0.895	54.5
Silicate-bound	0.875	31.8	0.316	16.4	0.259	15.8
Total	2.76	100	1.93	100	1.64	100
Bulk coal (μg/g)	4.00		2.43		2.00	
Recovery (%)	68.9		79.5		82.1	

Abbreviation: ND, not detected

**Figure 3:**
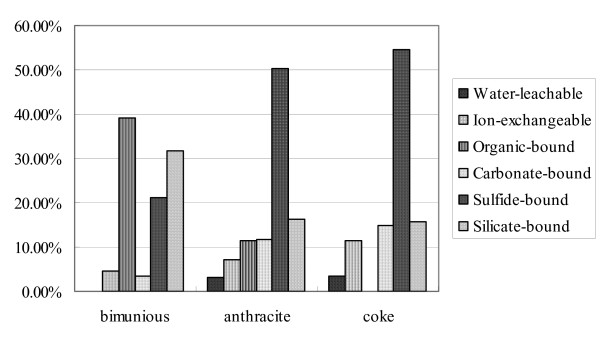
Histogram of fractions of Se forms determined by sequential extraction from three coals.

The amounts of selenium in each fraction extracted from the three coals are plotted in Figure 3.

#### (1) Water-leachable Se

The water-leachable Se is determined by refluxing the coal with double-distilled deionized water. During coal mining, storage, and transport, water-leachable Se is readily leached into the soil and ground water. The water-leachable Se in different ranks of coals of Huaibei coalfields are very low (Table 3) and the values are 0, 0.063, and 0.057 μg/g, respectively. In bituminous coal, water-leachable Se is lower than the detection limit, but is higher in anthracite and cokeite (3.23% and 3.45%, respectively, of the total).

#### (2) Ion-exchangeable Se

Ion-exchangeable Se in coal is soluble in a solvent such as ammonium acetate (NH_4_OAc). The ion-exchangeable Se in bituminous coal, anthracite and cokeite are 0.128, 0.138, and 0.186 μg/g, which are 4.66%, 7.14% and 11.34% of the total, respectively. In some previous studies, the water-leachable and ion-exchangeable Se are combined. In a study of Se in coal and overburden in the power River basin by [36], Se is present at the ppm level and occurs in at least six different forms. They found that the fraction of water soluble selenium in 14 overburden samples was 5–73%, with an average of 22%, of the total. Up to 50% of the selenium in the overburden samples was ion exchangeable (an average of 21%). Selenium was sorbed by clays and other phases in the overburden samples. Querol et al. studied the forms of Se in a subbituminous Spanish coal with an ash yield of 26.5% by sequential solvent extraction. The found that the fraction of water-soluble and ion-exchangeable Se was less than 10%[37]. Yang studied the abundances and modes of occurrence of trace elements in the Late Permian coals in the Puan Coalfield, Guizhou, China, and found that the water-soluble Se fraction was about 0.03%[38].

#### (3) Carbonate-associated Se

The carbonate-associated Se content in bituminous coal, anthracite and cokeite of Huaibei Coalfield is 0.093, 0.224, and 0.245 μg/g, which are 3.36%, 11.6%, and 14.9% of the total, respectively. The carbonate-associated selenium has previously been studied sequential extraction. Querol et al. found that the carbonate-associated fraction of Se was only less than 1%[37]. Yang reported that carbonate-associated Se fraction was about 0.33%. Their values are substantially lower than our results on Huaibei coals[38].

#### (4) Sulfide-associated Se

The sulfide-associated selenium in bituminous coal, anthracite, and cokeite is 0.581, 0.975, and 0.895 μg/g, which are 20.1%, 50.5%, and 54.5% of the total, respectively. The amount of sulfide-associated Se increases with increasing rank of coals. The sulfide-associated Se is the dominant mode of occurrence in anthracite and cokeite. Both selenium and sulfur are Group 6a elements in the Periodic Table with similar covalent radii and electronegativities. Selenium may have diffused from non-sulfide phases into pyrite during coal metamorphism. Selenides may also form in anthracite and cokeite.

Previous studies have found a positive correlation between Se and S. Palmer and Lyons analyzed some European and American coals and found that pyrite has a higher Se content than illite, kaolinite, and quartz[39]. Zhang et al. found that the Se content of pyrite in anthracite from southwestern Guizhou is significantly higher than that in calcite and clay minerals. Statistical analysis of 24 samples of typical coals from main British coal basins shows that pyrite is the main carrier of Se [40,41]. A positive correlation between Se and S in the three anthracite samples from the i3B seam in the eastern Donbas suggests that Se is associated with pyrite [42]. Other studies also suggested that Se and S are highly correlated in coal and Se mainly occurs in pyrite from several coalfields [43-48]. Some researchers have reported sulfide-associated selenium by sequential extraction. Liu performed a sequential extraction experiment to identify the forms of Se in coal as part of their study on volatilization of lignite, bituminous coal and anthracite[48]. The forms of selenium were determined as ion-exchangeable, organic matter-bound, sulfide-associated, and in residues. They showed that the sulfide-associated fraction of selenium accounts for 77% in lignite, 65% in bituminous coal, and 67% in anthracite. Yang has reported that sulfide-associated fraction of Se was 57.7%. Thus, the sulfide-associated selenium is the dominant mode of occurrence in coals[38].

#### (5) Organic matter-associated Se

The organic matter-associated Se in bituminous coal, anthracite and cokeite samples from Huaibei Coalfield is 1.08, 0.219, and 0 μg/g, respectively. The organic matter-associated fraction accounts for 39.1%, 11.3% of total Se in bituminous coal and anthracite, respectively (Figure 3). There is no organic matter-associated selenium in cokeite. When bituminous coal is converted to anthracite during metamorphism, the chemical structure of organic matter is changed, and selenium in the organic fraction may largely diffuse into other phases, most likely sulfides. Further, when anthracite is metamorphosed to cokeite, all selenium in the organic matter is incorporated into sulfides.

Other studies also showed that the organic matter-associated Se is a major mode of occurrence of Se in coal. Xu et al. performed sequential extraction tests and found that more than 70% of Se is associated with the organic matter[49]. Zhu et al. studied the modes of occurrence of Se in the Se-rich black shale in Yutangba area, Hubei, China, and found that Se resides dominantly in the residues and organic matter along with other forms of sulfide, selenide, and elemental Se[15,50,51]. Dreher and Finkelman reported that 10–20% of Se is in organic association in overburden sediments from a surface mine in Power River Basin, U.S.A[36].

#### (6) Silicate-associated Se

The silicate-associated Se content in bituminous coal, anthracite and cokeite samples from the Huaibei Coalfield is 0.875, 0.316, and 0.259 μg/g (Table 3), which are 30.2%, 16.4%, and 15.8% of the total, respectively (Fig. 3). The silicate-associated Se fraction in our Huaibei samples are higher than the literature results, which may be related to the influence of magmatic intrusions in the Huaibei Coalfield.

Dreher and Finkelman reported that the silicate-associated fraction of Se was about 15% of the total in overburden sediments of a surface mine in Wyoming, U.S.A.[36] Zhang et al. found that silicate-associated Se is less than 1%[40]. Palmer et al. found that their samples of U.S. coals have about 15% silicate-associated Se fraction [52]. Yang determined abundances and modes of occurrence of trace elements in the Late Permian coals in the Puan Coalfield, Guizhou, China, and found that the silicate-associated fraction of Se was about 3.81% of the total[38].

## 4. Conclusion

(1) We have performed sequential extraction tests to determine the forms of Se in three coals of bituminous, anthracite, and cokeite rank in the Huaibei Coalfield, Anhui, China. The Se content in bulk samples are 4.0, 2.4, and 2.0 μg/g in bituminous coal, anthracite, and cokeite, respectively. The Se content decreases with increasing rank of coals, which suggest that selenium may be partially lost during metamorphism.

(2) The results of six-step sequential extraction experiments show that the distribution of selenium forms varies with the rank. The fractions of water-leachable, ion-exchangeable, carbonate-associated and sulfide-associated selenium increase with increasing rank. On the contrary, organic-bound and silicate-associated Se decreases with increasing rank.

(3) The predominant form of Se in bituminous coal is organic matter-associated (39.1%), sulfide-associated (21.1%), and silicate-bound (31.8%). These three forms of Se account for 92% of the total.

The sulfide-associated fraction is 21.1%, 50.4%, and 54.5% of total Se in bituminous, anthracite and cokeite, respectively. Sulfide-bond selenium is much higher in anthracite and cokeite than in bituminous coal, indicating the formation of sulfide and selenide minerals during metamorphim.

The organic matter-associated fraction accounts for 39.1% and 11.3% of total Se in bituminous coal and anthracite, but it is totally depleted in cokeite. During metamorphism, organic matter-associated selenium in bituminous coal diffuses into other phases, most likely sulfides, in anthracite and cokeite.

### Acknowledgements

This work was supported by the National Natural Science Foundation of China (40772095) and the Anhui Natural Science Excellent Youth Foundation . We thank reviewers for constructive comments.
